# Transformation of remnant algal biomass to 5-HMF and levulinic acid: influence of a biphasic solvent system

**DOI:** 10.1039/d0ra02784g

**Published:** 2020-06-29

**Authors:** Liisa K. Rihko-Struckmann, Olalekan Oluyinka, Aditya Sahni, Kevin McBride, Melanie Fachet, Kristin Ludwig, Kai Sundmacher

**Affiliations:** Max Planck Institute for Dynamics of Complex Technical Systems Sandtorstr. 1 D-39106 Magdeburg Germany rihko@mpi-magdeburg.mpg.de; Otto-von-Guericke University Magdeburg, Universitätsplatz 2 D-39106 Magdeburg Germany

## Abstract

The primary commercial product from the green microalgae *Dunaliella salina* is β-carotene. After extracting the lipophilic fraction containing this red-orange pigment, an algal residue remains. As the carotenogenesis is induced by light stress with simultaneous nitrogen depletion, the protein content is low and the remnant is comprised largely of storage carbohydrates. In this work, we transformed the defatted remnant directly to the platform chemicals, 5-hydroxy methyl furfural (5-HMF) and levulinic acid (LA), without previous purification or any pretreatment. The batch experiments were carried out in an autoclave under biphasic solvent conditions at 453 K for 1 h using acidic ZSM-5 zeolite as a heterogeneous catalyst. Mixtures of methyl isobutyl ketone (MIBK/H_2_O) or tetrahydrofuran (THF/H_2_O/NaCl) with water were used to create the biphasic reactor conditions. The biphasic reaction mixtures helped to increase the 5-HMF yield and simultaneously mitigated the formation of insoluble humins. The carbon yields of 5-HMF and of LA in the MIBK/H_2_O biphasic system without NaCl were 13.9% and 3.7%, respectively. The highest carbon yield of 5-HMF (34.4%) was achieved by adding NaCl to the reaction mixture containing THF/H_2_O. The experimentally measured partition ratios of 5-HMF between the two liquid phases were compared to the predictions calculated by the computational method COSMO-RS, which is a quantum chemistry-based method to predict the thermodynamic equilibria of liquid mixtures and the solubilities. The COSMO-RS predicted partition ratios of 5-HMF were in line with the experimentally measured ones.

## Abstract 2

Defatted algal remnant is transformed to 5-HMF and LA.

## Graphical abstract

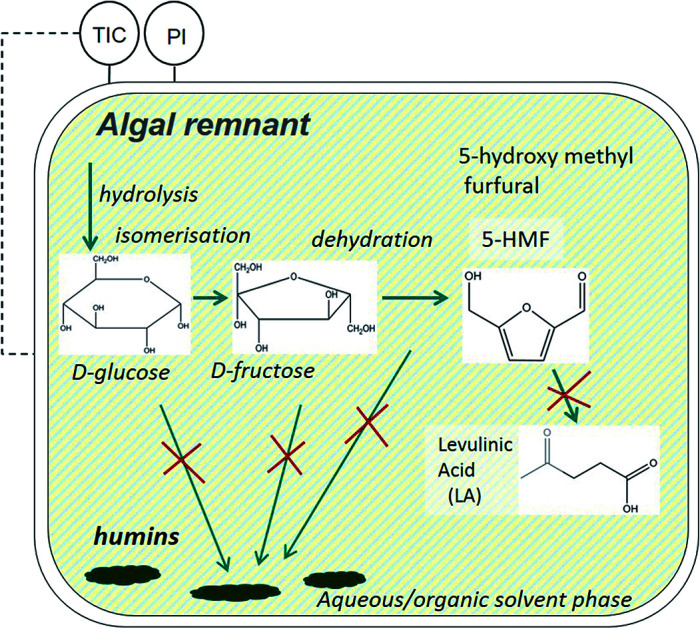

## 1. Introduction

Lignocellulosic biomass resources are considered to be largely available for the future production of chemicals and fuels.^1^ Transformation of biomass to renewable polymers, chemical building blocks, fuel additives, and liquid fuels reduces our dependence on non-renewable fossil resources and consequently reduces global emissions of greenhouse gases. 5-Hydroxy methyl furfural (5-(hydroxymethyl)furan-2-carbaldehyde, 5-HMF) and levulinic acid (4-oxopentanoic acid, LA) are excellent examples of biomass-derived, initial platform chemicals that can serve as versatile precursors for the production of a wide range of chemicals and liquid fuels.^2^ 5-HMF is a valuable intermediate for the production of 2,5-furandicarboxylic acid (FDCA), a potential renewable substitute for fossil based petrochemicals such as terephthalic and adipic acids. It also shows potential as a monomer for the production of polyethylene furanoate (PEF), a renewable polymer that exhibits high gas diffusion barrier properties for oxygen, water and carbon dioxide.^3^ LA is a renewable building block for the synthesis of many chemicals that have a high potential for industrial applications.^4^

The transformations of processed carbohydrates, *i.e.* starch, cellulose, or fully purified sugars, are being intensely investigated as biomass-based sources for the production of 5-HMF.^5^ Microalgae provide a highly promising alternative to the lignocellulosic, or sugar-based biomass sources due to their unique carbon concentrating photosynthesis mechanism (CCM), that efficiently assimilates CO_2_ leading to high biomass growth rates. Furthermore, microalgae have some additional advantages compared to terrestrial biomass sources. They can be cultivated in brackish or contaminated waste water or even under saline conditions in open ponds, thereby avoiding direct competition for agricultural land.^6^ The cell wall is free of lignin which facilitates its disruption prior to the fractionation and transformation of the algal macrobiomolecules, *e.g.* the carbohydrates. Efficient valorization of all biomacromolecules (lipids, pigments, proteins and polysaccharides) is the key to reach the economic viability microalgae production processes and to contribute to the sustainability goals.^7^

*Dunaliella salina* (*D. salina*), a unicellular biflagellate green algae, is a viable feedstock for producing valuable products such as glycerol and carotenoid pigments, mainly β-carotene.^8^ It is a photosynthetic, autotrophic organism capable of harnessing solar energy to produce biomass. The extraction of target oils and carotenoid pigments leaves behind a remnant biomass that is rich in carbohydrates and lean in protein. Much of this carbohydrate-rich remnant biomass can be hydrolyzed under mild temperatures to form monomers such as glucose and fructose.^9^

Numerous experimental findings convince us today, that biphasic reaction conditions are favorable for achieving high conversions of sugars, *i.e.* fructose to 5-HMF.^10,11^ MIBK, THF or 1-butanol are the most commonly selected solvents to constitute the organic phase in biphasic systems for 5-HMF synthesis.^12^

Heterogeneous catalysts are highly preferred for biomass conversion because of the simple separation of the products. For the conversion of sugars and the upgrading of lignocellulosic biomass-derived chemicals, zeolites have been reported to be highly suitable. However, one challenge in using zeolites in aqueous solutions is their stability.^13^ Zeolites have been largely investigated for the dehydration of sugars (fructose or glucose) toward 5-HMF and LA;^13–16^*e.g.* the beta topology,^14^ L-type^15^ and MFI-structured zeolites have been reported to mention a few. The ZSM-5 (MFI structure) has a three dimensional medium size pore system, and is available with a wide Si/Al ratio. The ZSM-5 type zeolite allows the diffusion of small molecules which results in shape and size selectivity. Importantly, the ZSM-5 has been demonstrated to be highly stable in deionized water at 150 and 200 °C for more than 6 h.^17^

The transformation of the microalgae remnant to 5-HMF includes the initial degradation of the microalgae cell structure, the hydrolysis of the polysaccharides (storage molecule starch) to glucose; and the isomerization of glucose to fructose before the final dehydration of fructose to 5-HMF. Wang *et al.* demonstrated the high functionality of the zeolite ZSM-5 for the reaction chain in a one-pot approach with *Chlorococcum* sp.^18^ The overall reaction chain includes Broensted and Lewis acid catalyzed reaction steps (Scheme 1 in ref. 19).

The stability of the catalyst in the presence of NaCl is of crucial importance in the biphasic systems of biomass conversion. Gardner *et al.* investigated the hydrothermal stability of ZSM-5 as a function of pH and the presence of NaCl in aqueous medium.^20^ High catalytic activity of ZSM-5 was observed after adding NaCl especially under acidic conditions. With pH 2.5 and saturating the solution with NaCl resulted in a glucose conversion of 63% and HMF selectivity to 33%. Importantly, Gardner *et al.* reported severe Al leaching in the presence of NaCl, and suggested that Al species in solution present Lewis acid properties required for the isomerization of glucose to fructose.^20^

The present contribution aims to accomplish the direct transformation of the defatted remnant of *Dunaliella salina* to 5-HMF and LA using the MFI structured ZSM-5 catalyst. Results of the transformation experiments under biphasic reactor conditions are presented. Preferably, a reliable predictive estimation of the phase equilibrium behavior is valuable prior to experimental measurements, as this allows one to reduce the amount of required lab work and facilitate the process design. Therefore, the experimental 5-HMF partitioning between the solvent phases are compared with COSMO-RS computational predictions.

## 2. Experimental

### 2.1. Materials and methods

*Dunaliella salina* biomass was purchased as a carotenoid-containing dry powder from Denk Ingredients GmbH, Germany (Art. No: 967996). Prior to hydrothermal treatment, the lipophilic compounds were extracted to obtain the defatted residual remnant biomass. Soxhlet extraction (6 h) with a ratio of 10 g biomass to 200 ml *n*-hexane (quality > 99%, Sigma-Aldrich) was used to simulate the industrial extraction of the nonpolar lipophilic molecules,^7,21^ carotenoids, and triglycerides from the delivered powder. The biomass remaining after lipid extraction was dried at 343 K overnight in an oven, cooled to room temperature in a desiccator, and used as remnant biomass in this study. The initial lipid content of the delivered biomass was estimated by weighing the lipophilic extract after the *n*-hexane removal in a rotavapor (see Table 1). Moisture and the ash content of the extracted remnant powder were determined by weighing the samples before and after overnight drying at 373 K and 723 K, respectively. The carbohydrate content was quantified using an enzymatic test kit (R-Biopharm AG, Germany) based on the UV-method^22^ and the method of Lowry^23^ was used for protein content determination. Table 1 shows the biomacromolecule composition of the defatted biomass and the initial lipid content of the delivered powder. In the defatted biomass, the sum of the proteins and carbohydrates is slightly below 100%. Due to the potential imprecision of the biomacromolecule determination for our samples, the content of elemental carbon was used as the calculation basis for the reported yields (see eqn (1)). In the *D. salina* defatted remnant, the carbon content was 43.2 ± 0.2 wt% measured with an automated elemental analyzer (Flash 200 Organic Elemental Analyser with MAS 200 R autosampler, Thermo Scientific, Germany).

**Table 1:** Characterisation of defatted *D. salina* powder^a^

Composition	wt%
Carbohydrates	85.6 ± 5.0
Proteins	8.46 ± 0.96
Moisture	1.26 ± 0.01
Ash	0.03 ± 0.002

a Lipid and carotenoid content in the biomass before extraction 11.5 ± 4.5 and 1.84 ± 0.1.

### 2.2 Catalytic transformation of the remnant biomass

The one-step chemical transformation experiments were carried out in a stainless steel reactor (Picoclave 3 autoclave, volume 200 ml, Büchi Labortechnik GmbH, Germany). 6 g of dried defatted remnant *D. salina* biomass (BM) were then mixed with ion free (Milli-Q, Merck, Germany) water or aqueous 23 wt% NaCl solution and solvent (MIBK (p.a.) or THF (p.a.)). The mass-based solvent phase/aqueous ratio was either 1 : 1, 1.5 : 1, or 2 : 1. The amount of water remained constant (50 g), and the amount of organic solvent (50, 75, or 100 g) used in the experiments is indicated in the illustrations. In the experiments with heterogeneous catalyst present, 1.00 g of the acidic zeolite catalyst H-ZSM-5 powder (CBV 3020E, module Si/Al ratio 35, pore diameter of 5.5 Å, Zeolyst Internationals) was added to the reaction suspension.

After placing the biomass, catalyst, and desired solvent mixture into the autoclave, it was purged with N_2_ for 10 minutes to flush out any air and then heated (about 10 min) to 453 K using a thermostat (Julabo SL-2) to circulate heating medium through the reactor jacket. The mixer (Büchi Glas Uster Cyclone 075) speed was fixed to 2000 rpm for all experiments. The pressure inside the reactor was recorded, but not controlled (autonomous). The reaction time was always one hour (60 min), whereas the starting time was considered to be the moment the temperature inside the reactor reached 453 K. After the desired reaction time, the reactor was cooled and vented before discharging. The solids were separated from the liquid phase product fraction (two phases at room temperature) by filtering with a weighed and dried filter paper (Whatman, *Ø* = 185 mm, 7–12 μm pore size). The solid products, a mixture of the unreacted algal remnant and formed humins were dried overnight at 343 K and weighed to estimate the overall remaining solid fraction. In the heterogeneously catalyzed experiments, complete recovery of the catalyst was assumed and the mass of the catalyst was subtracted from the solid weight before estimating the weight of the remaining solid fraction. In measurements with NaCl, the estimation of the remaining solids was not possible due to the partial crystallization of the NaCl, which contributed to the mass of the solid fraction to some extent.

The phase separation (organic/aqueous phase) of the filtrate was performed in a separating funnel at room temperature. After a settling period of 17 h, two distinct phases were separated and quantified by weight to determine the phase ratio. The phases were analyzed either by HPLC or by gas chromatography (see details below).

To assess the experimental uncertainty in the transformation experiments, three independent experiments were repeated under identical experimental conditions and the obtained standard deviation is depicted as error bars in the illustrations.

### 2.3 Aqueous and organic phase analysis

The 5-HMF and LA contents of the aqueous phase were analyzed with an HPLC (Agilent Hi-Plex H with RI detector, sample injection volume 20 μL) operating at 333 K. The mobile phase in the HPLC was 0.005 M H_2_SO_4_ with the flow rate of 0.6 ml min^−1^. Standard solutions with a range of different weight fractions were prepared for 5-HMF (≥99%, Sigma Aldrich) and LA (≥99%, Sigma Aldrich) in water and linear calibration correlations were obtained for both. After the phase separation, the amount of 5-HMF and LA in the aqueous phase was quantified with the obtained calibration equation.

The organic phase was analyzed using a GC/MS (Agilent 6890N) equipped with an HP-5 column (30 m, diameter 0.32 mm, film 0.25 μm). The analysis conditions were the following: He carrier gas: 1 ml min; injector: split mode (1 : 10), 523 K, 2 bar (overpressure); column temperature: from 398 K with a rate of 8 K min^−1^ to 418 K (26 min holding time), followed by a rate of 2 K min^−1^ to 493 K (10 min holding time). The compounds were identified using the NIST MS Search 2.0 database. The GC/MS detector signal was calibrated with standard samples of 5-HMF and LA, and linear calibration correlations were obtained.

### 2.4 Carbon yield of 5-HMF and of LA

The carbon yield of 5-HMF and LA were calculated using eqn (1):1
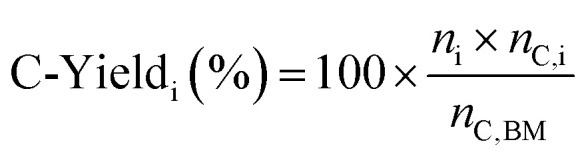
where the *n*_i_ is the amount (mol) of product i, i = 5-HMF or LA, *n*_C,i_ is the carbon amount in i (mol_C_ mol^−1^): (i = 5-HMF, *n*_C_ = 6) or (i = LA, *n*_C_ = 5). The carbon amount *n*_C,BM_ (0.216 mol_C_ in all experiments) was calculated from the measured elemental carbon content in the dried algae remnant biomass (43.2 wt%). The yield in biomass transformations can be reported variously: either the mass of the desired chemical product is expressed in relation to the total mass of the dried biomass,^24^ or the carbon amount in the product is expressed in relation to the feed carbon content or to carbohydrate amount in the biomass. The first calculation basis (dry weight of biomass) does not consider the variation in the composition of the biomass, *i.e.* the varying ratio of protein and carbohydrate fractions in the biomass, and is therefore less preferred for assessing the biomass to chemicals yield. In the present contribution we use eqn (1) as done by He *et al.*^2^ Wang *et al.* reported the yield of 5-HMF in relation to the carbohydrate amount in the feed, which is not directly comparable with our results.^18^ In comparing the results, the reader should be aware of the different definitions for yield.

## 3. Computational method

The COSMO-RS model combines quantum chemical COSMO calculations with statistical thermodynamics in order to make predictions of the chemical potential. Basically, the charged surface of a molecule is determined and divided into many, smaller segments. These charged segments are assumed to interact with all of the other segments in the mixture, which can contain one or more components. The energetic differences between the segments are calculated using an interaction model that also accounts for dispersion forces and hydrogen bonding. The chemical potential of the molecule is calculated from the individual segment–segment interactions and a combinatorial term that accounts for shape and size effects. This allows one to make thermodynamic predictions without requiring experimental data. This is especially useful for complex molecules, such as those encountered in biomass, which are often difficult to model using other methods.

The 5-HMF, LA, H_2_O, MIBK, THF and NaCl data (COSMO result files) for the energy optimal conformers were obtained from the COSMOtherm TZVP database. The COSMO files include the geometry optimisation and the calculation of the screening charge density for all conformers and therefore no geometry optimizations were required. The commercial program COSMOtherm (Version C3.0, release 16.01)^25^ was used for the estimations of component partition between the phases. They were calculated with the liquid–liquid property in the presence of salts with the BP_TZVP_C30_1601 parameterisation in COSMO-RS.

## 4. Results

### 4.1 Solubilization of defatted *Dunaliella salina* remnant

**Fig. 1:**
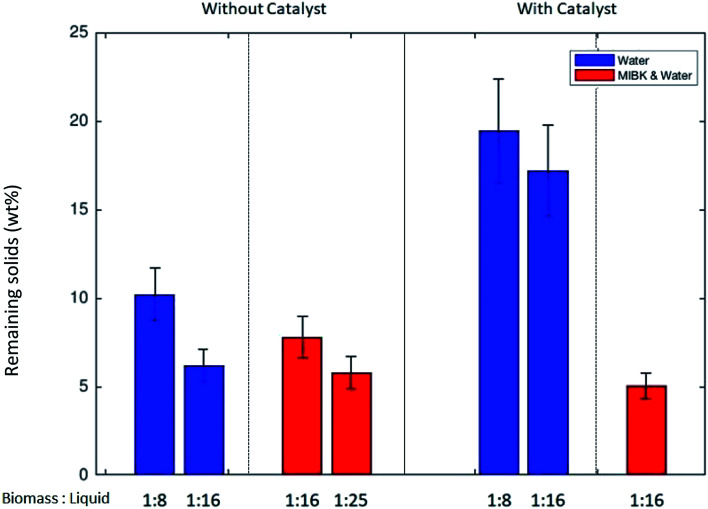
The remaining solids (wt%) in the experiments without (first four bars) and with the catalyst ZSM-5 (1 g) Zeolyst CBV 3020e. Biomass 6 g, the biomass/liquid ratio (w/w) noted in the bars, the blue bars with water as solvent, red bars with MIBK/H_2_O solvent mixture (1 : 1).

Fig. 1 illustrates the mass of remaining solids in relation to the loaded biomass (in mass %) after the transformation experiments without catalyst or under the influence of protonated zeolite catalyst. During the thermo-catalytic treatment at 453 K, the remnant fraction of *Dunaliella salina* was converted to solubles at a very high extent. Fig. 1 shows the resulting solids for the experiments carried out without the addition of NaCl. In experiments with high concentration of NaCl, the precise determination of remaining solids was not possible. The NaCl saturated liquid phase led to partial crystallization of NaCl, which contributes to the solid weight. Furthermore, one should note that both the residual, unconverted algal biomass and the macromolecular undissolved humins being formed during the liquefaction process contribute to the remaining solid fraction depicted in Fig. 1. A detailed characterization of the humin, a precise separation of the unconverted biomass, catalyst and humin, and a corresponding quantification of all the contributors to the solid fraction were outside of the scope of the present study.

Using an aqueous monophasic reaction medium, the presence of the ZSM-5 catalyst significantly influenced the amount of solids remaining (blue bars in Fig. 1). The amount of solids remaining in relation to the initial loaded biomass increased from 6.2 to 17.2 wt% by the inclusion of the acidic zeolite catalyst (BM/H_2_O ratio of 1 : 16). Comparable findings were also observed with the higher BM/H_2_O ratio (1 : 8). Based on these experimental findings, it is assumed that the acidic catalyst facilitated the humin formation by promoting the self-condensation of 5-HMF. As reported by van Zandvoort *et al.*,^26^ it is likely that the humins are formed due to cross-condensation of 5-HMF and LA. In addition to the self-condensation, the 5-HMF and glucose degradation are possible reaction paths for humin generation during the thermal treatment of biomass feeds containing carbohydrates.^27^ Structurally, humins are polymer networks of various oxygen containing functional groups.^26^ Noticeably, the catalyst increased the yield of LA and promoted the side reactions (soluble and insoluble humins) leading to deep brown coloration of the reaction mixture.

The biphasic reactor conditions with MIBK as the organic solvent (BM/MIBK/H_2_O ratio of 6/50/50 (w/w/w)) significantly increased the conversion of the remnant biomass to solubles while mitigating the amount of remaining solids (see Fig. 1, red bars). In the presence of organic and aqueous phases, it is likely that the humin formation is mitigated because 5-HMF has a high solubility in MIBK. Humins might be soluble in MIBK as well which would further lower the remaining solid fraction. Furthermore, we observed that when using the biphasic MIBK/H_2_O solvent mixture the amount of remaining solids was less dependent on the presence of catalyst. As seen in Fig. 1, the remaining solids in the biphasic system with MIBK remained below 8 wt% in all experiments.

### 4.2 Conversion of defatted algae biomass to 5-HMF and LA

**Fig. 2:**
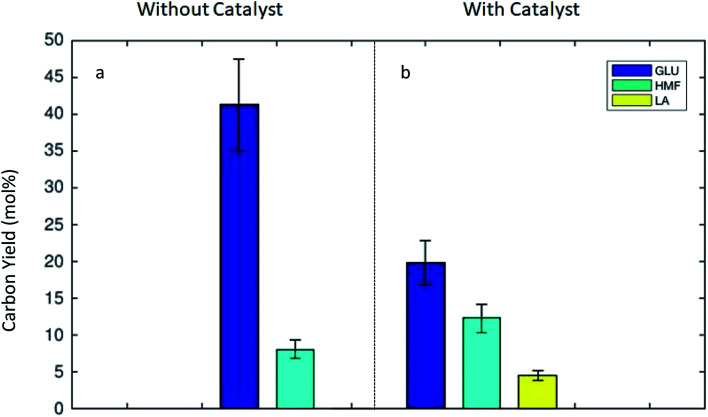
Carbon yield (%) to glucose (dark blue), 5-HMF (turquoise) and LA (yellow) in the aqueous monophasic system, (a) without catalyst, (b) with catalyst ZSM-5 (1 g) Zeolyst CBV 3020e, reaction time 60 min at 453 K. Biomass 6 g, BM/H_2_O ratio 1/8 (w/w).

The concentrations of the product molecules 5-HMF and LA in the organic and aqueous phases were determined using gas and liquid chromatograph. The carbon yields of glucose, 5-HMF, and LA were calculated according to eqn (1). Negligible amounts of other soluble compounds were detected in the analysis, but were not quantified. Fig. 2 illustrates the influence of the catalyst on the carbon yield of glucose, 5-HMF, and LA obtained under the monophasic aqueous conditions. Here, the catalyst clearly enhanced the glucose conversion toward 5-HMF and LA. The carbohydrates in the defatted *Dunaliella* biomass were easily transformable under mild hydrolysis conditions. The algal carbohydrate fraction was readily hydrolyzed to monosugars. In the absence of the catalyst high amounts of glucose were observed (see Fig. 2a). The main soluble product was glucose with a C-yield of 41.2%. In the monophasic experiment with water, a C-yield of 5-HMF 8.1% and minimal LA yield (0.1%) were observed in the absence of any catalyst. This observed unproblematic hydrolysis to sugar intermediates agreed well with our previous observations, where thermally treated (273 K) defatted *Dunaliella* remnant was successfully used as a carbon source for the cultivations of *Chlorella vulgaris*, *Escherichia coli* and *Saccharomyces cerevisiae*.^9^ These microorganisms were able to use the released sugars from defatted algal remnant as a carbon source for the heterotrophic growth.

By using the zeolite as a heterogeneous catalyst during the thermal treatment, the monosugars were sequentially dehydrated and the defatted biomass was transformed to the preferred intermediates, 5-HMF (C-yield of 12.3%) and LA (C-yield of 4.6%) by a significant amount (see Fig. 2b). Unfortunately, the presence of catalyst did not only support the desired dehydration to chemical intermediates, but also simultaneously facilitated the humin formation. This was apparent due to the large remaining solid fractions (19.4 or 17.2% at BM/H_2_O ratios of 1 : 8 or 1 : 16 (w/w)) under the monophasic reactor conditions. In summary, in the presence of the acidic zeolite catalyst, the overall C-yield toward the preferred key molecules (5-HMF and LA) was 16.9%. This is a significant improvement when compared to performing the reaction without any catalyst where the overall C-yield of 5-HMF and LA was only 8.2%.

**Fig. 3:**
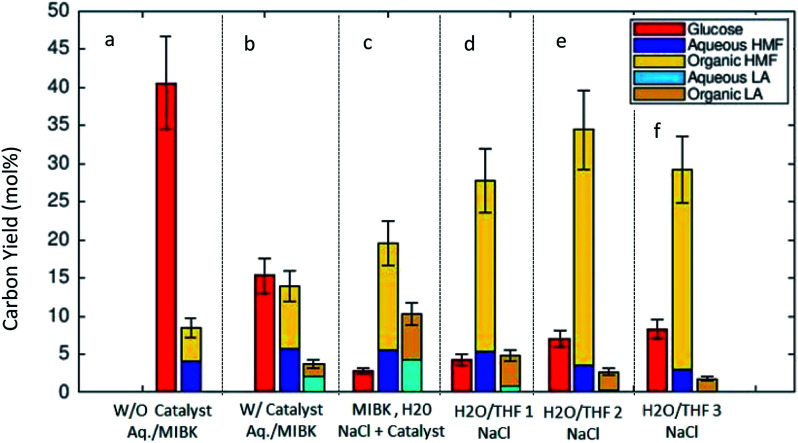
The C-yields [mol%] of glucose, 5-HMF and LA obtained in the experiments at 453 K and reaction time of 60 min. The distribution between the phases the organic and aqueous phases is indicated with legend colors. Glucose was detected only in the aqueous phase. (a) Without catalyst, MIBK/H_2_O (w/w) 50 : 50; (b) with catalyst, MIBK/H_2_O (w/w) 50 : 50; (c) with catalyst, MIBK/H_2_O/NaCl 50/50/14.9; (d) with catalyst THF/H_2_O/NaCl 50/50/14.9; (e) with catalyst THF/H_2_O/NaCl 75/50/14.9; (f) with catalyst THF/H_2_O/NaCl 100/50/14.9 (w/w/w).

Fig. 3 shows the results of the thermal transformation of algal remnant to glucose, 5-HMF, and LA under various biphasic reaction conditions. In the illustration, the C-yields of 5-HMF and LA are differentiated such that the partition of the compounds, 5-HMF and LA, between the two phases in the system is shown (solvent mixture MIBK/H_2_O in Fig. 3a/b/c and THF/H_2_O in Fig. 3d/e/f, respectively). For simplicity, the phases are labeled as organic and aqueous. Fig. 3a/b/c illustrate the C-yields of glucose, 5-HMF and LA using the MIBK/H_2_O reaction solvent mixture. In the absence of the catalyst, the algal carbohydrates were hydrolyzed, and glucose was detected in high concentrations in the aqueous phase. In the binary solvent mixture MIBK/H_2_O, the 5-HMF distributed balanced between the aqueous and organic phase. Without the catalyst, the C-yield of 5-HMF was 4.4% in the organic phase, and 4.0% in the aqueous phase (Fig. 3a). Fig. 3b–f show the C-yields for various biphasic systems in the presence of the acidic zeolite. By comparing the results in Fig. 3a and b (both with identical MIBK/H_2_O solvent compositions) one can see the clear effect the catalyst on the reaction. Under the influence of the heterogeneous acidic catalyst, the glucose dehydrates expectedly. In the MIBK/H_2_O mixture, the 5-HMF and LA formed are distributed amongst both phases. The C-yield of 5-HMF is 5.7% in the aqueous phase and 8.2% in the MIBK phase (Fig. 3b). Low concentrations of LA were detected in the heterogeneously catalyzed experiment with MIBK/H_2_O: the C-yields to LA (aq/org) were 2.1% and 1.6%, respectively.

It is known that the addition of NaCl clearly influences the 5-HMF distribution in the liquid–liquid equilibrium.^28^Fig. 3c shows the product distribution in the MIBK/H_2_O solvent mixture with a high amount of added NaCl (NaCl/H_2_O 0.30 (w/w)) in our experiments. The presence of NaCl changed the distribution of the target molecules (5-HMF and LA) towards the organic phase. 5-HMF concentrated in the organic phase, and a molar ratio, *n*_org_/*n*_aq_ of 2.56 (mol mol^−1^) for 5-HMF was measured for the solvent mixture comprised of an equally weighted mixture of MIBK/H_2_O (Fig. 3c). These findings corroborate the results reported by Mohammad *et al.*,^29^ where the effect of various electrolytes on the liquid–liquid equilibrium of MIBK/H_2_O were investigated. Furthermore, the salt presence influences the catalysis^20^ which was confirmed by us as well. Recently, an acceleration of initial 5-HMF formation from fructose or tagatose was reported under the influence of various salts in an aqueous sulfuric acid catalyzed system. The observed acceleration of the reaction rate in the homogeneous system was related to the anion quality of the salt.^30^ For zeolite catalysts, the salt inclusion might lead to leaching Al^+^ as reported by Gardner *et al.*,^20^ but this was not followed in the present study.

Fig. 3d–f illustrate the C-yields for a biphasic solvent system with tetrahydrofuran (THF) as the organic solvent. 5-HMF is highly soluble in THF, which in turn is miscible with water. When NaCl is included in the system, a biphasic liquid–liquid equilibrium is established. Fig. 3d–f report the measured carbon yields in the reaction mixtures with THF/H_2_O weight ratios of 1 : 1, 1.5 : 1, and 2 : 1, respectively. The C-yield of glucose increased slightly with increasing amounts of THF, but the C-yields of 5-HMF in the THF system were clearly higher than those of glucose or LA. Here, we achieved the highest C-yield in the organic fraction (30.9%) with a THF/H_2_O (w/w) ratio of 1.5 : 1. Another observation with the THF/H_2_O/NaCl system was that the formation of LA was suppressed, and the corresponding C-yield always remained below 5.0%. We obtained the best overall C-yield of the desired compounds, 5-HMF and LA (totally 37.1%), with a THF/H_2_O weight ratio of 1.5 : 1 (Fig. 3e). In addition to the reported experimental results shown in Fig. 1–3, we calculated by computational methods the corresponding estimations of the liquid phase compositions and the solubility of the target molecules. Their partitioning behaviors between the two liquid phases are discussed in more detail in Section 3.3.

**Fig. 4:**
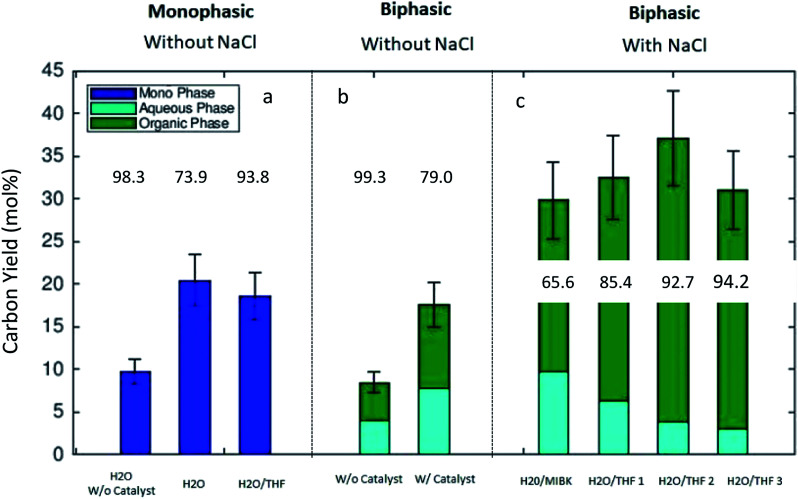
The overall carbon yield (5-HMF and LA) under (a) monophasic, NaCl free reaction conditions, (b) biphasic (MIBK/H_2_O) solvent system without NaCl and (c) biphasic solvent system with NaCl inclusion. The numeric values in the illustration give the product ratio (in %) *n*_5-HMF_/(*n*_5-HMF_ + *n*_LA_). Biomass 6 g, BM/solvent ratio 1/16 (w/w).

Fig. 4 summarizes the experimentally obtained C-yields (combined yield of 5-HMF and LA) in monophasic (a), NaCl-free biphasic (b) and NaCl containing biphasic (c) systems. The numeric values in Fig. 4 give the product ratio (in %) *n*_5-HMF_/(*n*_5-HMF_ + *n*_LA_). Without the addition of NaCl, the overall C-yield was measured under three monophasic conditions. Both monophasic systems (purely aqueous and THF/H_2_O) show comparable C-yields, 20.4% and 18.6%, with the catalyst. In a pure aqueous solvent without added catalyst, the yield remained low, and a C-yield of only 9.8% was obtained (Fig. 4a). In the NaCl-free biphasic system, the combined C-yield was comparable to those of monophasic systems both in the presence and absence of the catalyst. Without the catalyst, the yield was 8.5% and with catalyst a yield of 17.6% was obtained. Importantly, one should note that without NaCl (Fig. 4b), the partition behavior of the product molecules (5-HMF and LA) was balanced between the phases and no distinct advantage in the distribution between the two liquid phases was observed. The measured product distribution in the direct algal biomass transformation as carried out in the present study is well in line with the reported liquid–liquid-equilibria measurements of 5-HMF, water, and MIBK at 298.15 K.^31^

Fig. 4c presents the carbon yields for biphasic systems with the added electrolyte, NaCl. The addition of NaCl clearly influenced the transformation of the algal remnant as higher combined C-yields were obtained compared to biphasic reaction conditions without it. Furthermore, the partition of the 5-HMF and LA between the phases was distinctively influenced by the addition of the electrolyte. In the biphasic MIBK/H_2_O mixture the combined carbon yield increased up to 29.8% compared to that of only 17.6% without NaCl. The partition of the product between the phases was well in line with results published by Mohammad *et al.*^31^ A clear dependence of the partition coefficient of 5-HMF on the concentration of the added salt for extraction in MIBK/H_2_O system was reported and explained by ion pair forming in the aqueous solutions. For the solvent system THF/H_2_O, the influence of added electrolyte was even more distinctive. The addition of NaCl to the THF/H_2_O mixture led to the formation of two phases, and additionally, the presence of the electrolyte improved the combined C-yield and influenced on the partition of the product molecules between the phases. The main fraction of the 5-HMF was detected in the organic THF phase (Fig. 4c). Here, our findings with THF are in clear agreement with the results produced by Yang *et al.*^32^ where the THF/H_2_O biphasic system was investigated for the transformation of a purified sugar, fructose. They reported the 5-HMF yield of 71.5 mol% using the one-pot biphasic THF/H_2_O reactor system at 413 K. Considering the results in our investigation, we can conclude that the liquid–liquid equilibria established in the biphasic reaction system improved the C-yield of 5-HMF and LA in the direct algal remnant transformation. The experimental results presented in this work are very comparable with experimental findings found in the literature concerning the conversion of sugars, *e.g.* fructose, starch, or cellulose to 5-HMF and LA under biphasic reaction conditions.^33^ However, in most of the published studies, fructose, glucose, or cellulose are used after purification. In the present study, we have shown that the defatted remnant of *Dunaliella salina* algal biomass can be directly used for the production of chemical intermediates without intensive purification measures. The direct transformation to chemicals could give an added value for the low valued algal remnant after the main product β-carotene and neutral lipophilic compounds, *e.g.* triglycerides, are removed by extraction. Regarding the successful transformation of raw algal remnant as shown here, one should note that *Dunaliella salina* has exceptional weak cell wall (which gives for *D. salina* the unique ability to adapt to salinity variations) compared to other green algae species, *e.g. Chlorella vulgaris*. Furthermore, the remnant fraction is rich in carbohydrates because the main product, β-carotene is formed as a secondary metabolite explicitly under severe abiotic stress in the form of nitrogen nutrient depletion and excessive light.^9,24,34,35^

### 4.3 Computational estimation of algal biomass transformation and the partitioning of 5-HMF

A further goal in this study was to assess the practicality of using computational methods to make thermodynamic predictions of a system originating from real biomass. This is naturally challenging, since reliable computational estimations for such systems are not readily available. However, they can potentially enable one to perform high throughput solvent screening which could reveal more about the role the solvent takes in the reaction chemistry^12,36,37^ and deliver a deeper understanding of the catalytic pathways at the molecular level.^38^ Recently, the favorable kinetic effects gained when using mixed solvent systems in homogeneous catalysis for biomass derived oxygenates were analyzed with computational methods.^39^ On the other hand, computational approaches may well illuminate the role that the Al in ZSM-5 (ref. 40) or the effect of chloride ions have on biomass dehydration.^41^ In the present contribution, we report the experimentally observed beneficial effects of bi-solvent systems on the acidic catalyzed reaction of remnant biomass to the valuable intermediates 5-HMF and LA. Furthermore, we compared here the experimentally measured and computationally estimated partitioning behavior of the product molecule 5-HMF under aqueous/organic biphasic conditions. There are numerous studies that investigated the phase equilibrium of 5-HMF in various mixtures, but these are typically done using pure chemicals for phase equilibrium experiments under precisely defined conditions. Such ideal experimental conditions are expected to differ clearly from the reaction and phase separation conditions in our experimental algal remnant transformations. Our goal here is to investigate whether a relative correlation between the experimental observations and computational predictions can be made.

As described in the methods section, after the thermal transformation was complete, the reactor effluent was cooled to 298 K and the liquid phase was separated from the remaining solids (unconverted biomass and the catalyst). Two liquid phases formed at 298 K, which were analyzed along with the composition of the organic compounds. The thermal treatment of the algal remnant under multiphase reactor conditions generates a complex reactor effluent, containing not only the desired products, 5-HMF and LA, but also biomass cell debris, partially soluble macromolecular fractions, and unspecified humin. However, if indicative computational predictions could be obtained correlating satisfactorily with the phase partitioning behavior of the desired molecules when considering a real biomass decomposition mixture, one would gain valuable information applicable in the initial process design and optimization.

Experimental measurements of the ternary and quaternary systems containing 5-HMF in various aqueous-organic biphasic systems and binary interaction parameters have been reported in the literature for the activity coefficient models NRTL and UNIQUAC.^31,42^ However, fully predictive computational liquid–liquid estimations could preferably simplify and speed up the process design for biomass transformation by reducing the need for experimentally determined binary interaction parameters. To this end, the liquid–liquid equilibria and the partition coefficient of 5-HMF and LA between the two phases were estimated by the predictive computational method COSMO-RS (conductor-like screening model for real solvents)^43,44^ which requires only the structural information of the molecules.

The COSMO approach has been shown to make good predictions for 5-HMF partition in ethanol–, MIBK–water mixtures,^24^ and even for homogeneous catalyst partitioning in various solvent mixtures.^45^ Recently, COSMO-RS was used in a framework for solvent selection in the extraction of carotenoids from biomass.^46^

**Fig. 5:**
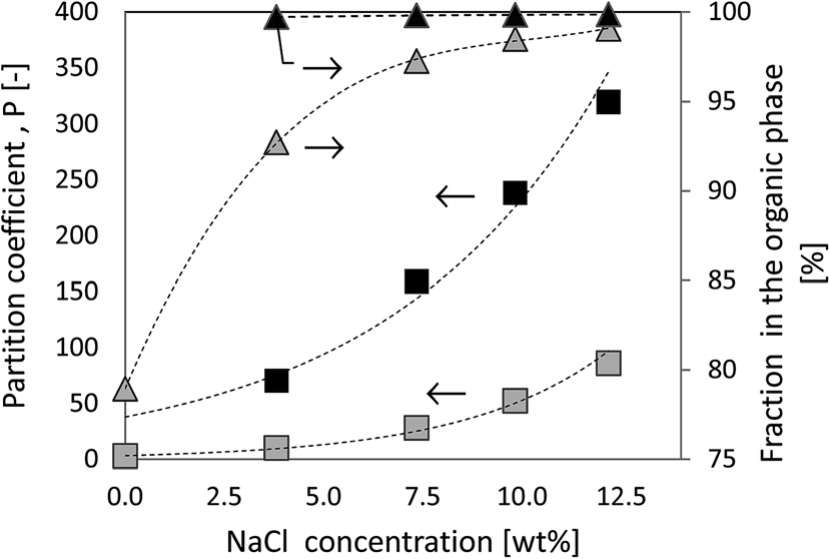
The COSMO-RS predicted partition coefficients, *P*, of 5-HMF under various solvent mixtures as a function of NaCl concentration. Grey symbols refer to MIBK/H_2_O mixture, black symbols to THF/H_2_O. The mass ratio of solvent/H_2_O/5-HMF is 50/50/1.

In the present study, the partition coefficients of the product molecule 5-HMF in a biphasic solvent mixtures consisting of MIBK/H_2_O and THF/H_2_O were estimated. The computational method allows one to make predictions how the NaCl concentration influences the partition of 5-HMF between the organic and aqueous phases. Fig. 5 illustrates the predictions made using COSMO-RS. The calculated weight fraction based partition coefficient, *P* as a function of NaCl (0.0 to 12.2 wt% NaCl) is calculated according the eqn (2):2
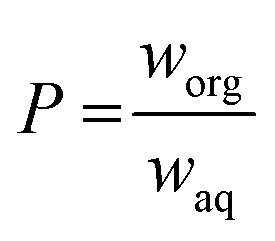
for a mixture containing equal mass amounts of either MIBK or THF and water. As seen in Fig. 5, the results provided by COSMO-RS clearly indicate that the addition of salt to the system influences directly the partitioning behavior of 5-HMF by increasing the fraction in the organic phase. In a mixture of MIBK/H_2_O/5-HMF (with w/w/w ratio of 50/50/1) without NaCl, the partition coefficient, *P*, achieves a value of 2.92, indicating a slightly higher weight fraction of 5-HMF in the organic phase; the concentrations of 1.39 wt% and 0.48 wt% in the organic and aqueous phase were expected by COSMO-RS calculations. By introducing NaCl to the MIBK/H_2_O mixture, COSMO-RS estimated the *P* value to increase strongly. With a NaCl content of 9.8 wt% (corresponding to MIBK/H_2_O/NaCl/5-HMF mass ratios of 50/50/11/1) COSMO-RS predicted a *P* value of 5-HMF to be 52.3 (98.5% of the 5-HMF would be in the MIBK rich phase), indicating a much higher concentration of 5-HMF in the organic phase compared to the absence of NaCl.

When using THF as the organic solvent, the predicted partition coefficient of 5-HMF is more pronounced. Here, COSMO-RS estimates the partition coefficient, *P* = 238 for 5-HMF with 9.8 wt% NaCl (with the mass ratios (w/w/w/w) 50/50/11/1 for THF/H_2_O/NaCl/5-HMF). This implies that 99.8% of 5-HMF would be located in the THF rich phase when liquid–liquid equilibrium is established. One can conclude from the COSMO-RS estimations that one could expect 5-HMF to distribute unevenly between the phases, preferably concentrating in the organic phase.

A comparison between the experimental measurements and the predicted values are summarized in Table 2. Here, the measured molar distributions (*n*_org_/*n*_aq_) for the 5-HMF in several biphasic reaction conditions along with those calculated using COSMO-RS are provided. As seen in Table 2 the experimentally measured values correlate qualitatively very well with the predicted ones. However, the predicted molar distributions (*n*_org_/*n*_aq_) of 5-HMF between the organic and aqueous phase were systematically higher than the experimentally measured distributions. This is in line with the recent review,^12^ where they reported a tendency of COSMO-RS to overestimate the presence of 5-HMF in the organic phase with various solvents. They suggest that the deviation might originate from the reacting systems, which does not only contain 5-HMF but the presence of unreacted sugars or undesired side-products might affect the distribution of coefficients. This is the most likely reason also in our investigations where biomass was directly used as feed instead of purified sugars. However, we can conclude that the relative order of the experimental results are well in line with the COSMO-RS predictions.

**Table 2:** The measured and COSMO-RS estimated amounts (*n*_org_/*n*_aq_) and molar fractions (*x*_org_/*x*_aq_) of 5-HMF in the organic and aqueous phases

Entry		*n*_org_/*n*_aq_. (exp.)	Relative (exp). [%]	Relative COSMO-RS [%]	*x*_org_/*x*_aq_ (exp.)	Relative (exp.) [%]	Relative COSMO-RS [%]
1^a^	MIBK/H_2_O	1.43	58.8	79.1	1.55	60.8	76.7
2^a^	MIBK/H_2_O/NaCl	2.58	72.1	91.7	3.78	79.1	93.0
3^a^^,^^d^	MIBK/H_2_O/NaCl	2.29	69.6	91.7	3.27	76.6	93.0
4^a^	THF/H_2_O/NaCl	4.13	80.5	97.3	8.33	89.3	97.6
5^b^	THF/H_2_O/NaCl	8.57	89.6	98.2	9.63	90.6	97.6
6^c^	THF/H_2_O/NaCl	8.88	89.9	98.7	7.50	88.2	97.6

a 1 : 1 organic solvent/H_2_O.

b THF/H_2_O (w/w) 1.5.

c THF/H_2_O (w/w) 2.0.

d Reaction time 2 h.

## 5. Conclusions

The algal, defatted remnant of *Dunaliella salina* was successfully transformed to the chemical intermediates 5-HMF and LA under monophasic and biphasic reactor conditions in the presence of the acidic zeolite catalyst ZSM-5. The solvent mixture strongly influenced the yield, and it was seen that by using a biphasic solvent system, the yield of 5-HMF was improved. The highest C-yield of 5-HMF (34.4%) was achieved with solvent mixture comprised of THF/H_2_O with the addition of NaCl to the reaction system in order to establish biphasic conditions. The measured molar distribution of 5-HMF between the two liquid phases was compared to computational predictions made using COSMO-RS. The relative order of the computational predictions is well in line with the experimental results, although the predictions overestimated the distributions towards the organic phase compared to those of experimentally measured. Despite this, our study provides strong motivation for further both computationally based as well as experimental investigations into solvent and solvent mixture design to improve further the yields of algal remnant to valuable chemical intermediates.

## Conflicts of interest

There are no conflicts to declare.

## Supplementary Material
